# An outbreak caused by the SARS-CoV-2 Delta variant (B.1.617.2) in a secondary care hospital in Finland, May 2021

**DOI:** 10.2807/1560-7917.ES.2021.26.30.2100636

**Published:** 2021-07-29

**Authors:** Iivo Hetemäki, Sohvi Kääriäinen, Pirjo Alho, Janne Mikkola, Carita Savolainen-Kopra, Niina Ikonen, Hanna Nohynek, Outi Lyytikäinen

**Affiliations:** 1 Infectious Diseases Unit, Kanta-Häme Central Hospital, Hämeenlinna, Finland; 2 Translational Immunology Program, Helsinki University and Helsinki University Central Hospital, Helsinki, Finland; 3 ECDC Fellowship Programme, Field Epidemiology path (EPIET), European Centre for Disease Prevention and Control, (ECDC), Stockholm, Sweden; 4 Finnish Institute for Health and Welfare, Helsinki, Finland

**Keywords:** SARS-CoV-2, COVID-19, VOC, health care associated infection, infection control

## Abstract

An outbreak caused by the SARS-CoV-2 Delta variant (B.1.617.2) spread from one inpatient in a secondary care hospital to three primary care facilities, resulting in 58 infections including 18 deaths in patients and 45 infections in healthcare workers (HCW). Only one of the deceased cases was fully vaccinated. Transmission occurred despite the use of personal protective equipment by the HCW, as advised in national guidelines, and a high two-dose COVID-19 vaccination coverage among permanent staff members in the COVID-19 cohort ward.

The severe acute respiratory syndrome coronavirus 2 (SARS-CoV-2) Delta variant of concern (Phylogenetic Assignment of Named Global Outbreak (Pango) lineage designation B.1.617.2) has been suggested to be more transmissible than the Alpha (B.1.1.7) variant [1], which is more transmissible than the wild-type SARS-CoV-2 virus [2-4]. We describe here an outbreak caused by the Delta variant that originated from one inpatient in a secondary care hospital and spread within the hospital and to three primary care facilities; we describe our experiences in controlling it. Cases were detected among patients, healthcare workers (HCW) and in the community. Both symptomatic and asymptomatic infections were found among vaccinated HCW, and secondary transmission occurred from those with symptomatic infections despite use of personal protective equipment (PPE).

## Setting and outbreak onset

Tavastia Proper healthcare district (HD), with a population of 171,000, is one of the 20 geographically and administratively defined HD in Finland. This HD has one central hospital providing secondary care and six healthcare facilities providing primary care, one of which is a district hospital while the other five are healthcare centre wards. The central hospital has 184 beds in eight wards and an intensive care unit (ICU). In the central hospital, patients who have symptoms compatible with coronavirus disease (COVID-19) are tested upon admission with a nasopharyngeal SARS-CoV-2 real time reverse transcriptase (RT-PCR) test (Supplementary data). Patients with a high clinical suspicion of COVID-19 are treated in isolation until there are at least two negative tests 24 hours apart.

In early May 2021, a patient with COVID-19-associated pneumonia and travel history in Asia was hospitalised for 4 days in the COVID-19 cohort in Ward 1 of the central hospital. This index patient had a positive test for SARS-CoV-2 9 days before hospitalisation and was admitted to an isolation room. Six days after the discharge of the index patient, two secondary case-patients tested positive for SARS-CoV-2 in Wards 1 and 2 (Figure). Exposed roommates (n = 8) and unvaccinated healthcare workers (n = 11, 10 of whom were HCW students) were quarantined. As additional cases were detected, Wards 1, 2 and 3 were closed from new admissions. All patients were treated with contact and droplet precautions three days following the identification of the first secondary cases.

**Figure:**
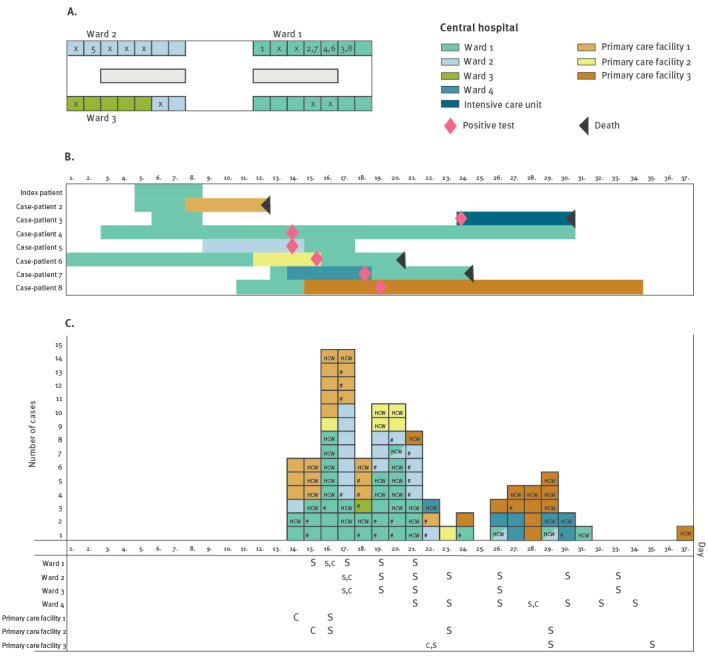
Outbreak caused by the SARS-CoV-2 Delta variant from one infected index patient in a central hospital, Tavastia Proper healthcare district, Finland, May 2021 (n = 103)

C: ward closure; COVID-19: coronavirus disease; HCW: healthcare worker; S: ward screening; SARS-CoV-2: severe acute respiratory syndrome coronavirus 2.

A) Start of the outbreak in Ward 1 (COVID-19 cohort), 2 and 3 in the central hospital. The rooms of the index case (1), case-patients 2-8 and other case-patients (> 8, marked with x) are indicated.

B) Hospitalisations in the central hospital and transfers to primary healthcare facilities for the index patient and case-patients 2–8 during the outbreak. A pink diamond indicates the date of SARS-CoV-2 laboratory confirmation and the black triangle indicates the date of death. The index patient had a SARS-CoV-2-positive test several days before hospital admission. Case-patient 2 was not laboratory-confirmed for SARS-CoV-2 before death, but had symptoms and transmitted the disease to a family member, healthcare workers and a roommate, who were all laboratory-confirmed for SARS-CoV-2.

C) Epidemic curve showing case-patients and HCW-cases by date when a positive SARS-CoV-2 sample was taken and place of exposure. Case-patients whose diagnosis was made after discharge from the exposure ward are marked with #. The date of diagnosis was missing for three HCW-cases in Primary care facility 1. Three HCW-cases who had worked both in Wards 1 and 2 are marked accordingly.

The outbreak spread to four wards in the central hospital. According to the place of exposure, most cases (31 case-patients and 21 HCW-cases) were in Wards 1 and 2, one case-patient was in Ward 3 and seven cases (four case-patients and three HCW-cases) were in another ward located on a different floor (Ward 4) (Figure). Exposure to SARS-CoV-2 occurred in almost every unit of the central hospital. Some exposed patients had been transferred to four of the six primary care facilities. In three of these four facilities, the outbreak spread through transfers that took place before the outbreak was detected. In the fourth facility, the transfer happened after outbreak detection and the exposed patient was quarantined and there was no further spread.

## Sequencing detection of the Delta variant

The sequencing results of the index patient as well as results of Case-patients 4 and 5, a staff member exposed to Case-patient 2, and several other (n = 32) outbreak-related samples showed that the outbreak was caused by the Delta variant (GISAID Accession IDs: EPI_ISL_2557176-EPI_ISL_2557210) [5]. 

In total, 317 laboratory findings positive for SARS-CoV-2 were notified to the National Infectious Diseases Register (NIDR) in Tavastia Proper HD in May 2021; 44% (141/317) were sequenced (NIDR, data taken 28 June 2021) and 41% (58/141) were the Delta variant, all but one with known transmission chains.

### Ethical statement

As the data displayed in this article is a result of an outbreak investigation (legal task of Finnish Institute for Health and Welfare and HD according to the Communicable Disease Act), ethical approval was not needed.

## Extent and spread of the outbreak

We defined a case-patient or HCW-case as a person with a positive SARS-CoV-2 RT-PCR test with a known exposure to a SARS-CoV-2 outbreak, either when admitted (patient) or when working in one of the four healthcare facilities (HCW).

In total, 58 case-patients were detected (Table 1), 18 of whom died. For the deceased case-patients, the median age was 80 years (range: 62–96), 11 were men, one was vaccinated with two doses, 11 with one dose and six were unvaccinated. For the majority of the deceased case-patients, COVID-19 likely contributed to their death. All had an underlying condition requiring hospital treatment prior to COVID-19 infection. There were 45 HCW-cases in four healthcare facilities (the central hospital and three primary care facilities; Table 1 ). There were no hospitalisations or deaths among the HCW-cases. Secondary infections (n = 62) occurred also in community in close contacts of case-patients and HCW-cases.

**Table 1:** Characteristics of COVID-19 case-patients and healthcare worker-cases, Tavastia Proper health district, Finland, May 2021 (n = 103)

Characteristics	Case-patients (n = 58)	HCW-cases (n = 45)	Total (n = 103)
Age in years (range)	73 (30–97)	38 (19–62)	-
Sex: men (%)	28 (48)	0 (0)	
Deaths (%)	18 (31)	0 (0)	
Vaccinated against SARS-CoV-2^a^			
Two doses	2	18	20
One dose	42	6	48
Unvaccinated	14	21	35
Place of exposure to SARS-CoV-2			
Central hospital^b^	36	26	62
Primary care facility 1	13	9	22
Primary care facility 2	3	3	6
Primary care facility 3	6	7	13

COVID-19: coronavirus disease; HCW: healthcare workers; SARS-CoV-2: severe acute respiratory syndrome coronavirus 2.

^a^ The vast majority of HCW (˃ 90%) were vaccinated with Comirnaty (BNT162b2 mRNA, BioNTech-Pfizer, Mainz, Germany/New York, United States).

^b^ Central hospital includes Wards 1-4.

At the time of the outbreak, 24 of 29 permanent HCW in Ward 1 were fully vaccinated with two doses of Comirnaty (BNT162b2 mRNA, BioNTech-Pfizer, Mainz, Germany/New York, United States), two of 29 had received one dose of Comirnaty when preceded by laboratory-confirmed SARS-CoV-2 within 6 months, two of 29 had received one dose of Comirnaty, and one of 29 was unvaccinated. In Ward 2, all (17/17) permanent HCW were fully vaccinated with Comirnaty. Vaccinations were given at a 3-week interval until mid-February 2021, and thereafter the interval was extended to 12 weeks. 

There were a few patients who contracted COVID-19 who had stayed solely in their single or two-person room and were cared for by fully vaccinated HCW (with universal masking), suggesting transmission from a vaccinated HCW-case. All staff members in Wards 1, 2 and 3 were screened for SARS-CoV-2 by RT-PCR the week after outbreak detection; screening in Ward 4 on the other floor was done the following week after the detection of secondary cases. Five asymptomatic infections were identified among fully vaccinated staff members; one developed symptoms only after the positive RT-PCR screening test (CT value: 17) and four remained asymptomatic (CT values: 28, 32, 33 and 34, suggesting low infectivity [6]). There was no secondary transmission from the four identified asymptomatic fully vaccinated HCW.

There was high vaccine coverage among permanent staff in the central hospital, but lower for HCW in primary healthcare facilities and among students in training and staff members who had no direct patient contact (Table 2). This is in line with the strict national COVID-19 vaccination strategy during a period of limited vaccine supply, which emphasised the prioritisation of HCW based on their statute and job description in order to have more vaccines available for elderly people and medical risk groups.

**Table 2:** Healthcare worker-cases and vaccination status by occupation, Tavastia Proper healthcare district, Finland, May 2021 (n = 45)

HCW occupation	Vaccinated^a^		Unvaccinated	Total
	2 doses	1 dose		
HCW with direct patient contact^b^	17	4	7	28
HCW students	0	0	8	8
Other staff^c^	1	2	6	9
Total	18	6	21	45

HCW: healthcare worker; SARS-CoV-2: severe acute respiratory syndrome coronavirus 2.

^a^ The vast majority of HCW (˃ 90%) were vaccinated with Comirnaty (BNT162b2 mRNA, BioNTech-Pfizer, Mainz, Germany/New York, United States).

^b^ Includes 2 HCW with one dose of vaccine and previous laboratory-confirmed SARS-CoV-2 infection.

^c^ Includes hospital cleaners and secretaries.

Among the fully vaccinated, symptomatic HCW-cases in the central hospital (n = 8), there were five cases for whom we had follow-up data unconfounded by other exposures to evaluate secondary transmission. Two HCW-cases with symptoms transmitted the infection to their household contacts and patients and one who also infected a HCW colleague within 4 days from the symptom onset. One HCW-case with symptoms transmitted the infection only to a household contact nearly 2 weeks after the disease onset of the HCW-case. Two HCW-cases did not infect anyone. We were not able to obtain complete data for the secondary transmission from all case-patients.

Of all the identified HCW-cases, five remained completely asymptomatic. The remaining HCW (n = 36, information missing for n = 4) had at least mild symptoms that, for a few, developed after screening. At the very beginning of the outbreak in Ward 1 and 2 and later in Ward 4, there was transmission that we could not trace; this could only be explained by infected HCW, suggesting that we most likely were not able to identify all HCW cases with mild or no symptoms. 

## Infection control measures

In the central hospital, COVID-19 patients are cohorted in Ward 1, which has 28 beds in 14 rooms. Four isolation rooms are equipped with negative pressure, while for the other 10 rooms, incoming air enters from a common supply line through a room-specific pipe and outgoing air exits through a room-specific pipe to a common exit line. The air supply and exit lines for Ward 1 are separated from those of Ward 2 and Ward 3 located at the same floor. Some staff members, medical doctors, physiotherapists and cleaning staff are shared by Ward 1 and 2. 

HCW used PPE (visor, long sleeved apron, gloves and surgical mask) in COVID-19 patients’ care. FFP2/3 respirators were used in aerosol-generating procedures and intensive care. Surgical masks were used by HCW in all contexts with patient contact (i.e. universal masking). Visitors were only allowed if they were asymptomatic and they were advised in hand hygiene and surgical mask wearing. Patients were encouraged to use surgical masks.

## Discussion

Since the start of the COVID-19 pandemic and as of 7 July 2021 in Finland, the cumulative number of laboratory-confirmed COVID-19 cases was 97,049 and that of COVID-19-related deaths was 976 (population: 5.5 million inhabitants). Before the outbreak described here, the 14-day incidence for laboratory-confirmed SARS-CoV-2 infections was 57 per 100,000 inhabitants for Tavastia Proper HD, and 52 cases per 100,000 inhabitants in the total Finnish population (source: NIDR). During the two weeks preceding the outbreak, 30% (856/2,848) of SARS-CoV-2-positive samples in Finland were sequenced (Supplementary data); of these, 57 were consistent with the Delta variant. Up to this point, there had only been one case of SARS-CoV-2 Delta variant in Tavastia Proper HD with a known transmission chain and no secondary transmission. Of note, at the start of the outbreak, vaccination coverage was the same in Tavastia Proper HD as in all of Finland i.e. 35% for the first dose and 4% for the second dose. The majority of those vaccinated were in the older age and medical risk groups.

This outbreak, which was caused by the SARS-CoV-2 Delta variant and led to 18 deaths in elderly people, occurred in four healthcare facilities despite the use of PPE, increasing vaccine coverage and universal masking by HCW. The technical department of the hospital did not deem possible that ventilation could explain the outbreak. Direct and indirect contact transmission are not considered to have an important role in SARS-CoV-2 transmission, compared to droplet transmission [7]. Pre-symptomatic yet infectious COVID-19 patients with varying incubation periods made it difficult to contain transmission both in the community and healthcare settings. It is possible that transmission occurred from asymptomatic/pre-symptomatic HCW to patients and other HCW. Although the first case-patients were diagnosed within 24 hours from symptom onset, the delay was enough for the outbreak to spread via patient transfer.

Breakthrough infections with the Delta variant and further transmission from fully vaccinated, symptomatic HCW occurred. Secondary transmission followed similar asymmetry as described with SARS-CoV-2 in unvaccinated individuals [8-10]. A recent study suggests reduced vaccine effectiveness of 36% against symptomatic disease caused by the Delta variant after one dose of Comirnaty vaccine [11], but excellent protection after full course; depending on disease severity, Comirnaty vaccine provides 88–92% protection against the Delta variant [11,12], and we saw similar rates among the fully vaccinated HCW in Ward 1.

To control the outbreak, surgical masks were replaced by FFP2 respirators when HCW are in close contact with a laboratory-confirmed COVID-19 patient, as supported by literature [10,13,14]. Previously FFP2/3 respirators were only used in aerosol-generating procedures and intensive care units. The Finnish national guidelines regarding use of PPE when treating patients with COVID-19 were changed accordingly based on ongoing discussion [15], even though we could not prove the airborne transmission during the outbreak. This was supported by the current good availability of FFP2 respirators.

In conclusion, this outbreak demonstrated that, despite full vaccination and universal masking of HCW, breakthrough infections by the Delta variant via symptomatic and asymptomatic HCW occurred, causing nosocomical infections. As the Delta variant continues to spread in Europe, we suggest that utilization of FFP2/3 respirators while treating COVID-19 patients should be included in national guidelines.

## Supplementary Data

86000 Supplementary Data
