# Supplementary material for: An outbreak caused by the SARS-CoV-2 Delta variant (B.1.617.2) in a secondary care hospital in Finland, May 2021
Source: Euro Surveill. 2021 Jul 29;26(30):2100636. doi: 10.2807/1560-7917.ES.2021.26.30.2100636 (PMC8323455; doi:10.2807/1560-7917.ES.2021.26.30.2100636)
Supplement: Supplementary Data [file 21-00636_HETEMAKI_SupplementaryData.pdf]

This supplementary material is hosted by Eurosurveillance as supporting information alongside the article "An outbreak caused by the SARS-CoV-2 Delta variant (B.1.617.2) in a secondary care hospital in Finland, May 2021" on behalf of the authors who remain responsible for the accuracy and appropriateness of the content. The same standards for ethics, copyright, attributions and permissions as for the article apply. Eurosurveillance is not responsible for the maintenance of any links or email addresses provided therein.

Supplementary Data

Clinically validated nasopharyngeal SARS-CoV-2 real time reverse transcription polymerase chain reaction (RT-PCR) test were performed in Fimlab laboratories (Tampere, Finland) with commercial RT-PCR kits by GenomEra®, Abacus Diagnostica; Cobas 8800®, Roche Diagnostics, and Allplex™, Seegene and in Synlab laboratories (Helsinki, Finland) by TaqPath, Thermo Fisher Scientific. Sequencing was done by Fimlab laboratories using Clean-plex SARS-CoV-2 FLEX-panel by Paragon Genomics (1) and the Finnish Institute for Health and Welfare using ARTIC primers (2, 3). The B.1.617.2 variants were assigned with the Pangolin COVID-19 Lineage Assigner (https://pangolin.cog-uk.io/, (4)). The strains had B.1.617.2 lineage defining spike mutations T19R, L452R, T478K and P681R and, in addition, deletion F157-R158 and mutations T95I, E156G and D614G.

COVID-19 cases are notified to the National Infectious Diseases Register (NIDR) by both laboratories and physicians. The laboratory notification also includes sequencing data.

References

1. PS7004-02_CleanPlex-SARS-CoV-2-FLEX-Panels.v3 [Internet]. [cited 28.6.2021].

2. COVID-19 ARTIC v3 Illumina library construction and sequencing protocol V.4 [Internet].; 2020 [updated May 29,; cited June 23, 2021]. Available from: https://www.protocols.io/view/covid-19-artic-v3-illumina-library-construction-an-bgxjjxkn?version_warning=no.

3. Quick Josh, Loman Nick, ARTIC Consortium. hCoV-2019/nCoV-2019 Version 3 Amplicon Set. 2020 Mar 24,.

4. Rambaut A, Holmes EC, O'Toole Á, Hill V, McCrone JT, Ruis C, du Plessis L, Pybus OG. A dynamic nomenclature proposal for SARS-CoV-2 lineages to assist genomic epidemiology. Nat Microbiol. 2020 Nov;5(11):1403-1407. doi: 10.1038/s41564-020-0770-5. Epub 2020 Jul 15. PMID: 32669681; PMCID: PMC7610519.
